# Rosiglitazone has a null association with the risk of prostate cancer in type 2 diabetes patients

**DOI:** 10.3389/fendo.2023.1185053

**Published:** 2023-07-25

**Authors:** Chin-Hsiao Tseng

**Affiliations:** ^1^ Department of Internal Medicine, National Taiwan University College of Medicine, Taipei, Taiwan; ^2^ Division of Endocrinology and Metabolism, Department of Internal Medicine, National Taiwan University Hospital, Taipei, Taiwan; ^3^ National Institute of Environmental Health Sciences of the National Health Research Institutes, Zhunan, Taiwan

**Keywords:** National Health Insurance, peroxisome proliferator-activator receptor gamma, pioglitazone, prostate cancer, rosiglitazone, thiazolidinediones

## Abstract

**Background:**

This study investigated the risk of prostate cancer in ever users and never users of rosiglitazone in diabetes patients in Taiwan.

**Methods:**

The nationwide database of the National Health Insurance was used to enroll male patients who had a new diagnosis of type 2 diabetes mellitus at an age ≥ 25 years from 1999 to 2005. A total of 11,495 ever users and 11,495 never users of rosiglitazone matched on propensity score were selected and they were followed up for the incidence of prostate cancer from January 1, 2006 until December 31, 2011. Cox proportional hazard model incorporated with the inverse probability of treatment weighting using the propensity score was used to estimate hazard ratios.

**Results:**

At the end of follow-up, incident cases of prostate cancer were found in 84 never users and 90 ever users of rosiglitazone. The calculated incidence was 173.20 per 100,000 person-years in never users and was 187.59 per 100,000 person-years in ever users. The overall hazard ratio (95% confidence intervals) for ever versus never users was 1.089 (0.808-1.466). The hazard ratios were 0.999 (0.643-1.552) for the first tertile (< 672 mg), 1.147 (0.770-1.709) for the second tertile (672-3584 mg) and 1.116 (0.735-1.695) for the third tertile (> 3584 mg) of cumulative dose. Sensitivity analyses consistently showed a null association between rosiglitazone and prostate cancer risk.

**Conclusion:**

Rosiglitazone has a null effect on the risk of prostate cancer.

## Introduction

Prostate cancer is the most common incident cancer in men over the world (1). It was estimated that there were 1.4 million cases of incident prostate cancer and 381,000 deaths ascribed to prostate cancer in 2016 (1). An increase of 40% in prostate cancer cases has been observed within the 10 years following 2006, probably because of the aging and growing population in the world (2). Incidence rates of prostate cancer are highest among the white people and lowest in the Asian populations, and may vary remarkably by 25-fold in different ethnicities (2). Although secular trend of prostate cancer shows a declining rate in the western world, the incidence of prostate cancer is increasing in Asian populations (3–6). Epidemiological data from Taiwan also show a steadily increasing trend in the incidence of (7) and mortality from (8) prostate cancer over the past decades. Although different times of adoption of prostate-specific antigen (PSA) as a screening tool in different countries may partly explain the discrepant trends observed in different ethnicities, genetic variations and changes in the prevalence of risk factors such as population aging and changes in dietary patterns with increasing rates of animal fat consumption and lifestyle changes with less physical activity and lack of exercise leading to obesity etc. are also possible explanations (2).

An increased risk of various types of cancer has been observed in patients with type 2 diabetes mellitus (T2DM). Although the mechanisms are not yet fully known, obesity, glycemic control, hyperinsulinemia, insulin resistance, comorbidities or antidiabetic drugs used to treat the patients are possible contributors (9–14). In contrary to a lower risk of prostate cancer being demonstrated in patients with T2DM in western countries (15, 16), a positive association in terms of mortality (8), incidence (7) and prevalence (17) has been observed in the Taiwanese population and in other Asian populations (18). A meta-analysis that includes 11 cohort studies conducted worldwide also supports that diabetes mellitus is associated with a significantly higher risk of all-cause mortality in patients with prostate cancer, prostate cancer-specific mortality and non-prostate cancer mortality (19).

Peroxisome proliferator-activator receptor gamma (PPARγ) is a nuclear receptor that functions as a transcription factor. Normal prostate and prostate cancer cells express PPARγ (20). Recent *in vitro* studies suggest that PPARγ agonists may play a dual role in the development and progression of prostate cancer (21). While the development and growth of prostate cancer can be inhibited by PPARγ agonists, stimulation of PPARγ may also directly lead to the carcinogenicity of prostate cancer via androgen receptor-dependent or -independent pathways (21).

Rosiglitazone and pioglitazone belong to a class of thiazolidinedione (TZD) and both have been used as antidiabetic drugs to treat hyperglycemia in patients with T2DM by improving insulin resistance via activation of PPARγ. However, rosiglitazone and pioglitazone may show different results in the association with cardiovascular disease and cancer in patients who use the drugs. For example, a suspicious bladder cancer risk has been reported for pioglitazone (22), but this was not observed for rosiglitazone (23). On the other hand, rosiglitazone has been shown to increase the risk of cardiovascular disease (24), but pioglitazone shows a beneficial effect (25). These discrepant pleiotropic effects of rosiglitazone and pioglitazone can be attributed to the different pathways influenced by different PPARγ agonists and the crosstalk between PPARγ and other signaling pathways (26).

In our previous study, pioglitazone shows a beneficial effect on prostate cancer risk after its prolonged use (27). However, whether rosiglitazone may exert a similar effect in humans has not been previously investigated. In *in vitro* studies using prostate cancer cell lines, rosiglitazone might inhibit the migration and invasion of prostate cancer cells through its inhibitory effect on the CXCR4/CXCL12 axis (28) and downregulation of vascular endothelial growth factor (29). PPARγ activation by rosiglitazone may also reduce the action of androgen receptor in androgen-dependent prostate cancer cells (30). In prostate cancer cell lines, rosiglitazone may affect cell cycle protein expression (31) and attenuate insulin-like growth factor 1 signaling (32). High rates of fatty acid and protein synthesis are required for the growth of prostate cancer cells, which may be blocked by the activation of fuel-sensing enzyme 5’-adenosine monophosphate-activated protein kinase (AMPK) (33). Although not consistently observed (34), metformin (a well-known activator of AMPK) reduces the risk of prostate cancer in Taiwanese patients with T2DM (35). Rosiglitazone has been shown to inhibit prostate cancer cell growth through its activation of the AMPK in both androgen-independent (DU145 and PC3) and androgen-sensitive (LNCaP) cells (33).

In humans, an early randomized placebo-controlled trial conducted in 106 patients with recurrent prostate cancer after radical prostatectomy and/or radiation therapy did not show any beneficial effect of rosiglitazone (4 mg twice daily) over placebo on the time to disease progression or posttreatment PSA doubling time (36). A meta-analysis suggests that TZD (including rosiglitazone and pioglitazone) has a null association with prostate cancer risk (37). However, another recent meta-analysis shows a null association between TZD use and prostate cancer risk in data derived from observational studies, but a significant risk reduction could be seen in data derived from randomized controlled trials (odds ratio 0.55, *P* = 0.04) (38).

Because rosiglitazone may show promising effects on prostate cancer cell lines but such a potential beneficial effect has not been extensively investigated in humans, this study was aimed to investigate whether rosiglitazone use might affect the risk of prostate cancer in patients with T2DM.

## Materials and methods

The government of Taiwan has implemented a unique, compulsory and universal health care system called the National Health Insurance (NHI) since March 1, 1995. The coverage rate of NHI is very high and includes > 99% of the population. Across Taiwan, all in-hospitals and 93% of all medical settings sign contracts with the Bureau of the NHI to provide healthcare services. According to local regulations, academic researchers can request for the use of the reimbursement database if the research proposal is reviewed and approved by an ethic review board. This study used the database after approval by the Research Ethics Committee of the National Health Research Institutes (approval number: NHIRD-102-175).

All personal data were de-identified for the protection of privacy. The International Classification of Diseases, Ninth Revision, Clinical Modification (ICD-9-CM) was used to code related diagnoses during the study period. Diabetes was coded 250.XX and prostate cancer 185.

**Figure 1:**
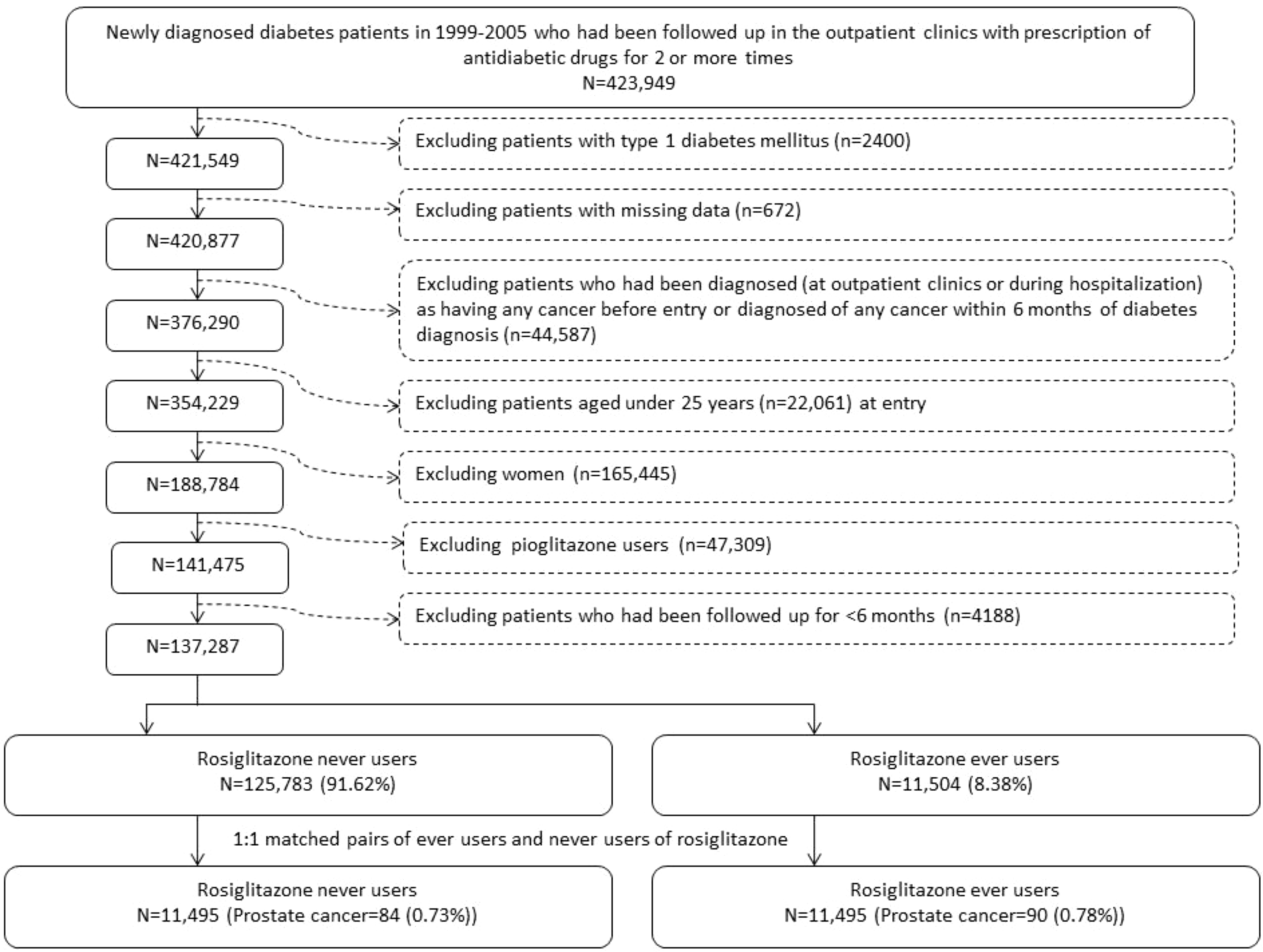
Flowchart showing the procedure in selecting a cohort of 1:1 matched-pairs of ever and never users of rosiglitazone based on propensity score into the study.

The selection procedures of a cohort consisting of 1:1 propensity score (PS) matched-pairs of rosiglitazone ever and never users from the NHI database are shown in **Figure 1**. The patients were newly diagnosed of diabetes mellitus from 1999 to 2005 and should have received antidiabetic drugs prescribed at the outpatient clinics for 2 or more times (n = 423,949). Patients who had a previous diagnosis of diabetes mellitus within 1996-1998 were not included to assure a new-onset of diabetes mellitus after 1999. The following patients were then excluded step by step: 1) type 1 diabetes mellitus (n = 2400, because rosiglitazone is not indicated for their treatment); 2) missing data (n = 672); 3) patients who had been diagnosed of any cancer (ICD-9-CM 140-208) before entry or within 6 months of diabetes diagnosis (n = 44,587); 4) age <25 (n = 22,061); 5) women (n = 165,445); 6) ever users of pioglitazone (n = 47,309); and 7) follow-up duration < 6 months (n = 4188). A cohort consisting of 1:1 matched-pairs of ever and never users of rosiglitazone was then created by the Greedy 8 ➔ 1 digit match algorithm based on PS (39). Logistic regression was used to create the PS from independent variables that included all characteristics listed in **Table 1** and the date of entry.

**Table 1:** Characteristics between never and ever users of rosiglitazone in a propensity score-matched cohort.

Variable	Never users (n=11495)		Ever users (n=11495)		*P* value	Standardized difference
	n	%	n	%		
Age* (years)	61.74	12.13	61.67	11.88	0.6323	-0.30
Diabetes duration* (years)	5.42	2.59	5.38	2.27	0.1699	-0.37
Hypertension	9518	82.80	9488	82.54	0.6012	-0.55
Chronic obstructive pulmonary disease	5644	49.10	5704	49.62	0.4287	1.13
Stroke	3842	33.42	3923	34.13	0.2587	1.63
Nephropathy	3647	31.73	3650	31.75	0.9661	0.14
Ischemic heart disease	5512	47.95	5562	48.39	0.5093	1.00
Peripheral arterial disease	3158	27.47	3167	27.55	0.8943	0.28
Eye disease	3999	34.79	4034	35.09	0.6283	0.85
Obesity	503	4.38	461	4.01	0.1670	-1.86
Dyslipidemia	9329	81.16	9373	81.54	0.4563	0.97
Benign prostatic hyperplasia	4292	37.34	4260	37.06	0.6624	-0.42
Urinary tract disease	6484	56.41	6543	56.92	0.4323	1.11
Statin	7740	67.33	7752	67.44	0.8659	0.29
Fibrate	5202	45.25	5292	46.04	0.2334	1.62
Angiotensin converting enzyme inhibitor/angiotensin receptor blocker	8659	75.33	8649	75.24	0.8785	-0.01
Calcium channel blocker	6613	57.53	6613	57.53	0.9999	0.15
Sulfonylurea	7683	66.84	7765	67.55	0.2494	1.41
Metformin	7938	69.06	8022	69.79	0.2292	1.43
Insulin	439	3.82	426	3.71	0.6523	-0.84
Acarbose	1094	9.52	1134	9.87	0.3725	1.08
Aspirin	7644	66.50	7632	66.39	0.8669	-0.08
Ticlopidine	727	6.32	752	6.54	0.5016	0.98
Clopidogrel	1638	14.25	1607	13.98	0.5571	-0.68
Dipyridamole	4549	39.57	4630	40.28	0.2754	1.61
Prostate-specific antigen	1656	14.41	1619	14.08	0.4851	-0.92

*Age and diabetes duration are expressed as mean and standard deviation.

In Taiwan, only rosiglitazone and pioglitazone in the class of TZD have ever been marketed. Users of pioglitazone were deliberately excluded in the analyses for the following reasons. Besides their glucose lowering effects, rosiglitazone and pioglitazone show different safety profiles in several clinical aspects. For example, rosiglitazone use has been shown to increase the risk of myocardial infarction and cardiovascular death in a meta-analysis (24). On the contrary, pioglitazone significantly lowers triglycerides and increases high-density lipoprotein cholesterol in a small clinical trial (40). Furthermore, pioglitazone reduces the risk of cardiovascular diseases in patients with T2DM (41) and reduces the risk of stroke and myocardial infarction in non-diabetes patients with ischemic stroke and insulin resistance (25). Our previous observational studies suggest a significantly lower risk of dementia associated with pioglitazone (42) but not with rosiglitazone (43). On the other hand, rosiglitazone significantly reduces the risk of breast cancer (44) and thyroid cancer (45), but pioglitazone shows a null effect on breast cancer (46) and thyroid cancer (47). Therefore, in the analyses of the safety profile and the risk association with cancer or non-cancer diseases, rosiglitazone and pioglitazone should be viewed as two different entities.

Age, diabetes duration, and factors that might be correlated with the exposure (rosiglitazone use) and/or the outcome (prostate cancer) in the study were considered as potential confounders (**Table 1**). These included hypertension (ICD-9-CM 401-405), chronic obstructive pulmonary disease (a surrogate for smoking; 490-496), stroke (430-438), nephropathy (580-589), ischemic heart disease (410-414), peripheral arterial disease (250.7, 785.4, 443.81 and 440-448), eye disease (250.5, 362.0, 369, 366.41 and 365.44), obesity (278), dyslipidemia (272.0-272.4), benign prostatic hyperplasia (600), urinary tract diseases (590-599), and use of the following drugs: statin, fibrate, angiotensin converting enzyme inhibitor/angiotensin receptor blocker, calcium channel blocker, sulfonylurea, metformin, insulin, acarbose, aspirin, ticlopidine, clopidogrel, and dipyridamole. The use of PSA test was also included because it may affect the detection rate of prostate cancer.

The differences for age and diabetes duration between never users and ever users of rosiglitazone were compared by Student’s t test and the other characteristics of categorical variables by Chi-square test. The value of standardized difference for each covariate was then calculated and a threshold value > 10% was used to indicate a potential confounding from the variable (48).

Cumulative dose of rosiglitazone was calculated in mg and a dose-response relationship was assessed by its tertiles. The incidence density of prostate cancer was calculated with regards to rosiglitazone exposure in subgroups of never users, ever users and the tertiles of cumulative dose of rosiglitazone therapy. The numerator was the number of incident prostate cancer diagnosed during follow-up. The denominator was the person-years of follow-up, which started on January 1, 2006 and ended up to December 31, 2011, at the time of a new diagnosis of prostate cancer, death or on the date of the last reimbursement record.

Hazard ratios that compared ever users to never users and compared the tertile subgroups of cumulative dose of rosiglitazone therapy to never users were estimated by Cox proportion hazard model incorporated with the inverse probability of treatment weighting using PS (49). Overall hazard ratios for ever versus never users of rosiglitazone were also estimated in the following sensitivity analyses after excluding: 1) patients who received a PSA test before the diagnosis of prostate cancer; 2) patients who had a diagnosis of any other cancers during follow-up; 3) patients having a diagnosis of benign prostatic hyperplasia; 4) patients with a diagnosis of nephropathy; 5) patients with a diagnosis of urinary tract disease; 6) patients with a diagnosis of benign prostatic hyperplasia, nephropathy and/or urinary tract disease; and 7) patients aged < 45 years.

SAS statistical software (version 9.4, SAS Institute, Cary, NC) was used as a tool for conducting all the statistical analyses. *P* < 0.05 was used as a threshold indicator for statistical significance.

## Results

The characteristics of the PS matched-pairs consisting of 11,495 never users and 11,495 ever users of rosiglitazone are shown in **Table 1**. All *P* values by Student’s t test and Chi-square test were > 0.05 and all variables had values of standardized difference < 10%, suggesting that the two groups were well matched on the covariates and residual confounding was unlikely.

**Table 2** shows the incidence of prostate cancer by rosiglitazone exposure and the hazard ratios comparing ever to never users and ever users categorized by the tertiles of cumulative dose to never users. There were 84 incident cases of prostate cancer in never users and 90 incident cases in ever users. The incidence rates in never users and ever users were 173.20 per 100,000 person-years and 187.59 per 100,000 person-years, respectively. The overall hazard ratio of 1.089 (95% confidence interval 0.808-1.466) suggests a null effect of rosiglitazone on prostate cancer. When examining prostate cancer risk by the tertiles of cumulative dose, none of the hazard ratios was statistically significant.

**Table 2:** Incidences of prostate cancer and hazard ratios by rosiglitazone exposure.

Rosiglitazone use	Cases followed	Incident cases	Person-years	Incidence rate (per 100,000 person-years)	Hazard ratio		95% Confidence interval	*P* value
Never users	11495	84	48499.58	173.20	1.000			
Ever users	11495	90	47977.54	187.59	1.089		(0.808-1.466)	0.5765

Cumulative dose (mg)								
Never users	11495	84	48499.58	173.20	1.000			
<672	3757	26	15173.16	171.36	0.999		(0.643-1.552)	0.9961
672-3584	3816	34	16964.46	200.42	1.147		(0.770-1.709)	0.4988
>3584	3922	30	15839.92	189.39	1.116		(0.735-1.695)	0.6063

The results of the sensitivity analyses are shown in **Table 3**. None of the hazard ratios reached statistical significance, supporting the null effect of rosiglitazone as observed in the main analyses (**Table 2**).

**Table 3:** Sensitivity analyses estimating overall hazard ratios for ever versus never users of rosiglitazone for prostate cancer.

Model	Hazard ratio	95% Confidence interval	*P* value
I. Excluding patients who had been screened by prostate-specific antigen before prostate cancer diagnosis	0.838	(0.518-1.355)	0.4706
II. Excluding patients with a diagnosis of other cancers during follow-up	1.085	(0.806-1.462)	0.5896
III. Excluding patients with a diagnosis of benign prostatic hyperplasia	0.854	(0.329-2.214)	0.7454
IV. Excluding patients with a diagnosis of nephropathy	1.133	(0.784-1.638)	0.5058
V. Excluding patients with a diagnosis of urinary tract disease	1.295	(0.698-2.404)	0.4119
VI. Excluding patients with a diagnosis of benign prostatic hyperplasia/nephropathy/urinary tract disease	1.293	(0.289-5.776)	0.7369
VII. Excluding patients aged <45 years	1.090	(0.809-1.469)	0.5699

## Discussion

The present study suggests a null effect of rosiglitazone on prostate cancer risk in patients with T2DM (**Tables 2**, **3**).

Unlike what has been observed in a previous study that pioglitazone may exert a beneficial effect on prostate cancer risk after a prolonged use (27), rosiglitazone seemed to have a null effect in the present study (**Tables 2**, **3**). It is interesting that these two drugs in the same class of TZD may exert different effects on cardiovascular disease (24, 25, 41) and on some types of cancer (22, 23, 37, 44–47). The crosstalk between PPARγ and other signaling pathways may probably explain the different clinical effects observed for different PPARγ agonists (26). Another explanation of a lack of protective effect of rosiglitazone is because of the lack of its effect on prostate cell growth at therapeutic levels of rosiglitazone used to treat diabetes. For example, an *in vitro* study showed that rosiglitazone at the therapeutic level of 1 μM did not affect prostate cell growth in cell cultures derived from normal, transformed or cancerous tissues (50). Even if there could be a minor beneficial effect of rosiglitazone on prostate cancer development, the body weight gain (obesity is a potential risk factor of prostate cancer (51)) commonly associated with rosiglitazone use might have attenuated such a minor beneficial effect (52). Because *in vitro* studies suggest that PPARγ agonists may exert dual effects on prostate cancer (21), the clinical impact of the use of rosiglitazone depends on the trade-off between these dual effects of PPARγ agonists.

Some *in vitro* studies suggest that excessive fatty acids may facilitate the malignant progression of prostate cancer promoted by PPARγ (53, 54). While pioglitazone may significantly reduce triglycerides (40, 54), rosiglitazone on the other hand would raise triglycerides (40, 54). Recent human studies suggest an association between triglycerides and prostate cancer risk (55, 56), severity (57) and recurrence (58). A recent *in vitro* study shows that the synthesis of lipid droplet and the proliferation and migration of prostate cancer cells activated by the PPARγ pathway can be effectively promoted by low-dose rosiglitazone (59). Whether the differential effects on lipid profiles between pioglitazone and rosiglitazone could explain their discrepant effects on prostate cancer risk awaits further confirmation.

The present study has some strengths to render good generalizability of the findings. First, the diagnoses from all claim records found at outpatient visits and hospital admission were included to reduce the possibility of missed diagnoses. Second, bias resulting from differential detection rates of prostate cancer because of different socioeconomic status could be much reduced because patients with a diagnosis of cancer can be waived for most medical co-payments by the NHI. Furthermore, the drug co-payments in patients with low income and in veterans are very low and the co-payments for patients who refill their drug prescriptions for chronic disease can be waived. Third, self-reporting bias could be reduced by the use of objective medical records.

There are some limitations in the study. First, actual measurement data were lacking in the database for potential confounders such as anthropometric factors, lifestyle, dietary factors, physical activity, smoking, alcohol drinking, hormonal profiles, family history and genetic parameters. Second, adhering to a healthy lifestyle including healthy weight, healthy diet, refraining from smoking and vigorous physical activity has been associated with a lower risk of developing prostate cancer in genetically predisposing men (60). However, it is recognized that most of the modifiable risk factors were not available in the database and their potential confounding effects could not be investigated. Third, this study investigated the effect of rosiglitazone on prostate cancer risk in patients with T2DM and without prostate cancer at baseline. Because some *in vitro* studies suggest an inhibitory effect of rosiglitazone on the growth of prostate cancer cells (28–33), additional research will be needed to look into the usefulness of rosiglitazone for the treatment of prostate cancer. Fourth, because of lack of information, the impact of biochemical data and the pathology, grading and staging of prostate cancer could not be evaluated.

In conclusions, this study suggests a null effect of rosiglitazone on prostate cancer in Taiwanese male patients with T2DM. Even though *in vitro* and animal studies may suggest a beneficial effect of rosiglitazone on prostate cancer cells, such a benefit cannot be readily applied to humans who use the drug for the treatment of T2DM. However, it is recognized that human studies are still sparse and therefore more studies are required to confirm the findings of the present study. TZD derivatives with more potent anticancer effects on prostate and breast cancer cells are being investigated for the potential development into anticancer drugs for the treatment of prostate cancer and breast cancer (61, 62). Because PPARγ activation may have a dual effect on prostate cancer (21), results derived from cellular studies should be carefully interpreted and clinical trials in humans are pivotal to elucidate the roles of different TZD compounds in the development or prevention of prostate cancer.

## Data availability statement

The datasets presented in this article are not readily available because public availability of the dataset is restricted by local regulations to protect privacy. Requests to access the datasets should be directed to C-HT, ccktsh@ms6.hinet.net.

## Ethics statement

The studies involving human participants were reviewed and approved by the Research Ethics Committee of the National Health Research Institutes. Written informed consent for participation was not required for this study in accordance with the national legislation and the institutional requirements.

## Author contributions

C-HT researched data and wrote manuscript. The author confirms being the sole contributor of this work and has approved it for publication.
